# HIV Knowledge Among Cisgender Female Sex Workers of Haitian Descent Working at the Border of Haiti and Dominican Republic

**DOI:** 10.3389/frph.2021.700861

**Published:** 2021-10-05

**Authors:** Henna Budhwani, Kristine R. Hearld, Julia Hasbun, John Waters

**Affiliations:** ^1^Department of Health Care Organization and Policy, School of Public Health, University of Alabama at Birmingham, Birmingham, AL, United States; ^2^Department of Health Services Administration, School of Health Professions, University of Alabama at Birmingham, Birmingham, AL, United States; ^3^Caribbean Vulnerable Communities Coalition (CVC), Kingston, Jamaica

**Keywords:** HIV, sex workers, Haiti, Dominican Republic, HIV knowledge

## Abstract

In this brief report, we aim to assess levels of HIV mis-information among cisgender Haitian female sex workers engaged in sex work at the Haiti and Dominican Republic border. We conducted bivariate analyses on the 2014 Border Study on Sex Workers comparing responses from female sex workers on the Haiti side of the border to those from their peers on the Dominican Republic side (*N* = 212). Prevention of HIV acquisition by correct and consistent condom use with each sex act was correctly endorsed by 90.5% of female sex workers in Haiti but only 57.0% of their peers in Dominican Republic (χ2 = 32.28, *p* < 0.001); 84.1% of respondents in Haiti correctly identified that HIV can be transmitted through a single unprotected sexual act, compared to 52.3% in Dominican Republic (χ2 = 25.2, *p* < 0.001). Significantly higher percentages of female sex workers in Dominican Republic correctly responded that HIV can be transmitted in pregnancy, compared to respondents in Haiti (96.5 vs. 71.4%; χ2 = 21.42, *p* < 0.001). Higher percentages of respondents in Dominican Republic correctly answered that HIV can be transmitted through needle sharing, relative to respondents in Haiti (100.0 vs. 89.7%; χ2 = 9.45, *p* < 0.01). Respondents in Dominican Republic more accurately rejected the possibility of transmission through food or through mosquito bites, compared to respondents in Haiti (95.4 vs. 81.8%, χ2 = 8.51, *p* < 0.01; 97.7 vs. 86.5%, χ2 = 7.81, *p* < 0.01, respectively). Findings indicate that if HIV knowledge is examined aggregating responses to individual questions, then elements of misinformation may remain unaddressed. For example, we found significant differences in correct answers ranging from 16.7 to 100.0%.

## Introduction

Promoting HIV knowledge-including the understanding of modes of transmission and prevention methods- to key populations, including commercial, cisgender female, and transgender female sex workers is at the foundation of HIV-related public health interventions and risk-reduction strategies (1–3). The Latin America and Caribbean (LAC) region has high rates of HIV, second only to Sub-Saharan Africa; female sex workers in the LAC region are at exacerbated risk for HIV due to their low socioeconomic status, stigmatized and powerless role in society, and residence in nations that criminalize sex work making it difficult if not impossible for victimized sex workers to seek protections though police and legal channels (4–6). Haiti is a low income country and, within the LAC region, holds the highest national rate of poverty, leading to some of its most vulnerable residents being forced into sex work to support their families (2, 7). In contrast, its island neighbor, Dominican Republic is a middle-income nation with notably higher financial standing (8). Haiti holds the highest rate of HIV in the western hemisphere with a generalized epidemic affecting 2–3% of its population, while Dominican Republic's epidemic is concentrated among key populations with <1% of its total population living with HIV (7, 8). The variability in poverty and rates of HIV have created a volatile situation in their shared island, with Haitian female sex workers soliciting clients on both sides of the Hispaniola border (5). Past research has established the precarious circumstances of those working at geographic borders, (6) but few studies have been able to capture data from matched populations of both sides of a given border. Thus, the purpose of this exploratory study is to examine and compare levels of HIV knowledge and mis-information among Haitian cisgender female sex workers engaged on both sides of the Haiti and Dominican Republic border.

## Methods

### Study Design and Participants

We performed descriptive analyses on the Border Study on Sex Workers. Data collection occurred from February 2014 to June 2014. Inclusion criteria were: (1) having exchanged sexual acts for commodities, including cash, in the past year, (2) being 18 years or older, (3) assigned female sex at birth, and (4) identifying of Haitian ethnicity (*N* = 212).

### Recruitment and Setting

Research Assistants recruited cisgender female sex workers at known hot spot sites on both sides on the Dominican Republic and Haiti border. These hots spots included street zones, sex work businesses, hotels with hourly rental policies, and bars. These hot spots are where non-governmental organizations routinely implement outreach and HIV testing efforts. After a potential participant was recruited, she was interviewed by a trained Research Assistant in these same community settings. Snowball sampling was employed to recruit participants wherein cisgender female sex worker participants could refer other potential peer participants from their social and professional networks; surveys were conducted in Spanish or Haitian Creole. Although the survey was available in French, no participants selected French.

### Ethics Review and Informed Consent

El Consejo Nacional de Bioética en Salud provided ethical approval for study design and data collection. Ethical approval for secondary analysis was obtained from University of Alabama at Birmingham's Institutional Review Board (#IRB-300001560). Due to the low literacy rates amongst this population, informed consent was obtained verbally. A trained Research Assistant read the IRB approved consent document to each participant in their preferred language; options included Spanish, Haitian Creole, or French. The potential participant was asked if they understood what was recited aloud, and then their verbal consent was requested via a yes to confirm participation or a no to decline participation. A fieldwork supervisor witnessed this process; all verbal consent procedures were approved during primary ethics review.

### Community Engagement in Survey Development

The survey questionnaire was co-developed by the Caribbean Vulnerable Communities Coalition research team along with peer outreach workers from the Centro de Promoción y Solidaridad Humana (CEPROSH), a local n-governmental organization based on the Dominican Republic side of the border. CEPROSH actively works with Haitian sex workers in the border areas. This collaborative approach ensured that the survey would be acceptable to cisgender female sex workers in this locale.

### Measures of HIV Knowledge

To ascertain level of HIV knowledge, respondents were asked if HIV could be transmitted through (1) sexual relations without a condom just once, (2) anal sex, (3) mosquito bites, (4) food sharing, (5) needle use, (6) pregnancy, (7) breastfeeding, and (8) public toilet. The ninth and tenth questions asked if respondents believed that individuals can protect themselves from HIV via proper condom use and if they think a person who looks healthy can have HIV.

### Statistical Analyses

Bivariate analyses (chi square and *t*-tests) examined differences between respondents engaged in sex work in Haiti and Dominican Republic. Chi-square tests were used to test associations between location of sex work and categorical variables and *t*-tests were used to test a difference in means of continuous variables by location of sex work. All statistical analyses were conducted using Stata 15.

## Results

Bivariate results are in Table 1; 40.6% reported working in Dominican Republic, and 59.4% reported working in Haiti. About 85% of respondents working in Haiti had some primary education, compared with 67% of respondents in Dominican Republic (χ2 = 11.57, *p* < 0.01). Prevention of HIV acquisition by correct and consistent condom use with each sex act was correctly endorsed by 90.5% of cisgender female sex workers in Haiti but only 57.0% in Dominican Republic (χ2 = 32.28, *p* < 0.001); 84.1% of the female sex workers in Haiti correctly identified that HIV can be transmitted through a single unprotected sexual act, compared to 52.3% of their peers in Dominican Republic (χ2 = 25.2, *p* < 0.001). Almost 93 (92.9%) of cisgender female sex workers in Haiti correctly rejected the notion of transmission through contact with a public toilet, compared with only 66.3% of peers in Dominican Republic (χ2 = 24.54, *p* < 0.001). Risk of acquisition of HIV from anal intercourse was correctly answered by 59.3% of cisgender female sex workers in Dominican Republic, but by only 16.7% of those in Haiti (χ2 = 41.43X, *p* < 0.001). Significantly higher percentages of cisgender female sex workers in Dominican Republic correctly responded that HIV can be transmitted transplacentally (in pregnancy), compared to peer sex workers in Haiti (96.5 vs. 71.4%; χ2 = 21.42, *p* < 0.001). Significantly higher percentages of cisgender female sex workers in Dominican Republic correctly answered that HIV can be transmitted through needle sharing, relative to their peer respondents in Haiti (100.0 vs. 89.7%; χ2 = 9.45, *p* < 0.01). Also, respondents in Dominican Republic more accurately rejected the possibility of HIV transmission through sharing food with a person living with HIV or through a mosquito bite, compared to cisgender female sex worker respondents in Haiti (95.4 vs. 81.8%, χ2 = 8.51, *p* < 0.01; 97.7 vs. 86.5%, χ2 = 7.81, *p* < 0.01, respectively).

**Table 1:** HIV knowledge held by cisgender female sex workers in Dominican Republic and Haiti (*N* = 212).

	**Dominican Republic *N* (%)**	**Haiti*N* (%)**	**χ2 / t**
Age (Mean/Standard Deviation)	28.68 (3.29)	27.12 (3.34)	**t** **=** **3.37** ***p*** **<** **0.001**
Education			
None Less than secondary completed Secondary	21 (24.42%) 58 (67.44%) 7 (8.14%)	10 (7.94%) 107 (84.92%) 9 (7.14%)	**χ2** **=** **11.57** ***p*** **<** **0.01**
Can a person protect themselves from HIV through correct condom use each time they have sexual relations?			
Yes No	49 (56.98%) 37 (43.02%)	114 (90.48%) 26 (9.52%)	**χ2** **=** **32.28** ***p*** **<** **0.001**
Can a person get HIV through having sexual relations without a condom just once?			
Yes No	45 (52.33%) 41 (47.67%)	106 (84.13%) 20 (15.87%)	**χ2** **=** **25.22** ***p*** **<** **0.001**
Can a person get HIV if they have only anal sex?			
Yes No	51 (59.30%) 35 (40.70%)	21 (16.67%) 105 (83.33%)	**χ2** **=** **41.43** ***p*** **<** **0.001**
Can a person contract HIV from mosquito bites?			
Yes No	4 (4.65%) 82 (95.35%)	23 (18.25%) 103 (81.75	**χ2** **=** **8.51** ***p*** **<** **0.01**
Can a person contract HIV by sharing food with an infected person?			
Yes No	2 (2.33%) 84 (97.67%)	17 (13.49%) 109 (86.51%)	**χ2** **=** **7.81** ***p*** **<** **0.01**
Can a person contract HIV by receiving an injection with a needle that was used before by another person?			
Yes No	86 (100%) 0 (0%)	113 (89.68%) 13 (10.32%)	**χ2** **=** **9.45** ***p*** **<** **0.01**
Can a pregnant woman infected with HIV transmit the virus to her unborn child?			
Yes No	83 (96.51%) 3 (3.49%)	90 (71.43%) 36 (28.57%)	**χ2** **=** **21.42** ***p*** **<** **0.001**
Can a woman infected with HIV transmit the virus to her newborn child through breastfeeding?			
Yes No	73 (84.88%) 13 (15.12%)	101 (80.16%) 25 (19.84%)	χ2 = 0.78 *p* = 0.38
Do you think that a person who looks healthy can have HIV?			
Yes No	54 (62.79%) 32 (37.21%)	92 (73.02%) 34 (26.98%)	χ2 = 2.49 *p* = 0.11
Do you think that you can get HIV by using a public toilet?			
Yes No	29 (33.72%) 57 (66.28%)	9 (7.14%) 117 (92.86%)	**χ2** **=** **24.54** ***p*** **<** **0.001**

*Bold indicates statistical significance*.

## Discussion

HIV research often includes scales of HIV knowledge to assess gaps and identify intervention targets; (9, 10) however, our findings indicate that if researchers, policy makers, and clinical providers exclusively examine HIV knowledge as a combination of answers to individual questions, then nuance may be lost, and serious elements of mis-information may persist among high-priority key populations. We found statistically significant differences across the levels of understanding in female sex workers in Haiti compared to their peers in Dominican Republic. Specifically, correct answers ranged from a low of 16.7% to the accurate response that one can contract HIV from anal sex by respondents in Haiti to a high of 100.0% to the accurate response that one can contract HIV through the use of pre-used needles by respondents in Dominican Republic, illustrating HIV prevention efforts that include an educational component, such as intervention development or public health programs, should target HIV knowledge gaps that are unique to local settings. Some of this work has already begun with prominent scholars questioning the psychometric properties of individual questions, in an effort to refine assessment of HIV prevention for certain key population (11). Lowest and highest rates of accurate responses may indicate successful or failed HIV prevention efforts, related to specific targets (e.g., prevention of maternal to child transmission, reducing needle sharing, etc.).

In this exploratory study, substantive trends emerged. We found that cisgender female sex workers in Haiti were more likely to correctly answer questions related to the protective value of condom use as compared to female sex workers in Dominican Republic, perhaps indicating that years of HIV prevention investments from the global development agencies have been successful in reducing mis-information related to condom use (12). In contrast though, only about 71% of female sex workers in Haiti knew that a woman living with HIV could infect her baby during pregnancy, which is especially worrisome, because Haiti is one of the few remaining countries in the western hemisphere that is still disproportionately affected by mother-to-child transmission (13). The notable rates of respondents who believed that someone who looked healthy could not be living with HIV (37.2% from respondents in Dominican Republic and 27.0% from respondents in Haiti) may allude to female sex worker's internalization of HIV-related stigma (14) associated with physical appearance, which could translate to increased risk, if these women are willing to engage in commercial sex work with partners who look healthy, and thereby in their estimation, may not be living with HIV. Thus, each of these individual questions highlighted disparities in understanding and pointed to, potentially, different intervention targets.

Limitations include the potential for biased responses due to lower literacy levels among cisgender female sex workers. Certain terms were not defined in the questionnaire. For example, we did not describe what is meant to “look healthy” in the context of the “Do you think that a person who looks healthy can have HIV” question, and therefore, responses to related such questions may have produced response variability due to differences in question interpretation. Our data was collected in 2014; HIV knowledge levels could have shifted over time, and may have improved due to the humanitarian efforts and programmatic outreach by the local government, international not-for-profit agencies, and regional groups that focus on HIV prevention. We leveraged snowball sampling; thus, the sample produced may be selective and subject to selection bias, and our study is cross-sectional and we only conducted bivariate analyses, so findings are not generalizable and we cannot make causal inferences.

## Conclusion

Our data is from one of the few repositories that contains information from cisgender female sex workers engaged on both sides of a geographic border and therefore provides a novel perspective on HIV knowledge and mis-information among this high priority, hard-to-reach, key population. Ensuring adequate HIV knowledge is critical to reaching the UNAIDS 90-90-90 targets in the LAC region, (15) but identifying noteworthy knowledge and behavioral goals may require nuanced approaches in which researchers examine disaggregated data to identify gaps and elucidate differences between geographically proximal key populations who may be affected by very different legal provisions, contexts, and public health programs.

## Data Availability Statement

The raw data supporting the conclusions of this article will be made available by request to the senior author on this paper, Dr. John Waters.

## Ethics Statement

El Consejo Nacional de Bioética en Salud provided ethical approval for study design and data collection. Ethical approval for secondary analysis was obtained from University of Alabama at Birmingham's Institutional Review Board (#IRB-300001560). Written informed consent for participation was not required for this study in accordance with the national legislation and the institutional requirements.

## Author Contributions

HB was the lead author. KH and HB conceptualized this study. JW was the senior author, and with JH conducted the original data collection and study formulation. KH was the lead methodologist. All authors contributed to the writing and editing of this manuscript.

## Funding

Research reported herein was supported by the University of Alabama at Birmingham (UAB) Sparkman Center for Global Health and the National Institute of Mental Health (NIMH) of the National Institutes of Health (NIH) under Award Number K01MH116737 (Budhwani).

## Author Disclaimer

The content is solely the responsibility of the authors and does not necessarily represent the official views of the National Institutes of Health or the UAB Sparkman Center for Global Health.

## Conflict of Interest

The authors declare that the research was conducted in the absence of any commercial or financial relationships that could be construed as a potential conflict of interest.

## Publisher's Note

All claims expressed in this article are solely those of the authors and do not necessarily represent those of their affiliated organizations, or those of the publisher, the editors and the reviewers. Any product that may be evaluated in this article, or claim that may be made by its manufacturer, is not guaranteed or endorsed by the publisher.
